# Porcine circovirus type 2 and its associated diseases in southwestern Nigeria: Farmers’ perception and level of awareness

**DOI:** 10.5455/javar.2022.i585

**Published:** 2022-06-26

**Authors:** Oluwawemimo Oluseun Adebowale, Olufemi Samuel Amoo, Kayode Olayinka Afolabi, Abimbola Adetokunbo Oloye

**Affiliations:** 1 Department of Veterinary Public Health and Preventive Medicine, Federal University of Agriculture, Abeokuta, Nigeria; 2 Centre for Human Virology and Genomics Research, Nigerian Institute of Medical Research NIMR, Yaba, Nigeria; 3 Molecular Epidemiology and Public Health Research Group (MEPHREG), Department of Biological Sciences, Anchor University, Nigeria; 4 Anchor University Center for Global Health (AUCGH), Nigeria; 5 Department of Veterinary Surgery and Theriogenology, Federal University Agriculture, Abeokuta, Nigeria

**Keywords:** Awareness, Pig farmers, Porcine circovirus type 2, Postweaning multisystemic wasting syndrome

## Abstract

**Objective:**

Porcine circovirus type 2 (PCV2) is one of the most important causative agents of swine diseases that pose a global economic threat. Presently, there is little or no information on the perception and awareness of PCV2 and its associated effects among pig farmers in Nigeria. Therefore, this research was carried out to describe pig farmers’ views, awareness, and likely impact of PCV2 and its associated postweaning multisystemic wasting syndrome (PMWS) on pig production in the southwestern region of Nigeria.

**Materials and Methods:**

A cross-sectional survey of pig farmers in Oyo and Ogun states, Southwest Nigeria, was carried out with the help of a self-administered questionnaire.

**Results:**

A total of 111 farms out of the 385 required took part in the study, resulting in a total response rate of 28.8%. 89 (79.2%, 95% CI = 70.8–85.8) pig farmers who participated were unaware of PCV2, while 46 (41.4%, 95% CI = 32.7–50.7) had heard about PMWS. The level of awareness was generally poor, with an average score of 1.43 (SD ± 1.25; 23.9%). Only 23% (25/111) of the participants had a high level of awareness. To promote awareness about PCV2/PMWS, participants’ most preferred sources of information were seminars, extension services (especially by veterinary and agricultural extension officers), social media (WhatsApp and YouTube), and mobile telephone (through calls or text messages).

**Conclusions:**

The present study showed a gap in the level of farmers’ awareness about PCV2/PMWS, and to bridge the gap, more scientific-based evidence is needed to promote targeted educational programs and policy formulations. Also, with the dearth of information about PCV2, it is necessary to determine its prevalence and the characteristics of the virus possibly circulating within the swine herds in Nigeria.

## INTRODUCTION

Porcine circoviruses (PCVs) belong to the *Circovirus* genus belonging to the family Circoviridae. Viruses of this group have single-stranded DNA genomes of about 1.76 kb, enclosed in a naked capsid [1]. The detection of PCV1 occurred as a contaminant of the cell line from a pig’s kidney (PK-15) in 1974, and further investigation into its virulence showed that it was nonpathogenic [2]. However, PCV2, detected about 20 years later in pigs with a systemic disease called postweaning multisystemic wasting syndrome (PMWS), has become a swine pathogen of huge economic importance globally [3,4]. Recently, another pathogenic species that has 55% and 37% identity (for replicase and capsid proteins, respectively) with PCV2 has been detected in the USA (designated as PCV3) from diseased pigs, having a bigger genome size of 2,000 nt compared to the previous two species [5]. PCV2 was initially implicated as the main causative pathogen of PMWS in weaned pigs and growers aged 6–16 weeks. In addition to PMWS, the virus has been linked to various swine diseases, generally categorized as porcine circovirus-associated diseases (PCVADs). Table 1 describes the porcine circovirus-associated diseases and their clinical features [4,6,7].

PMWS, as one of the PCVADs, is a multifactorial systemic swine disease of high economic importance in the pig industry [8]. In their investigation of the financial implications of PMWS in some farrow-to-finish facilities in England in 2008 before the large-scale vaccination, estimates of £52.6 million and £88 million per year were spent during the periods of no outbreak and when there was an outbreak, respectively. Considering the magnitude of the typical economic loss due to PMWS in the English swine industry, the need for rigorous surveillance on PCV2 and its other coinfecting pathogens cannot be overemphasized in any pig-producing nation in the world. However, to date, there has been gross neglect in determining the presence and prevalence of PCV2 in the swine of many countries that produce pigs in sub-Saharan Africa, including Nigeria [9].

In Nigeria, pig farming has been the source of livelihood for thousands of pig farmers struggling to meet the demand of over 50 million consumers, despite its great potential to grow [10]. While working to achieve the UN Sustainable Development Goals (SDGs 2 and 3), Nigeria’s falling nutrition levels and increasing undernourished people have put the country in the opposite position [11]. Therefore, the expanding nutritional deficit calls for increased livestock production and valorization of the value chains and supplies to meet the twin challenges of 1) the current artificially suppressed needs and 2) the rapidly increasing human populations’ animal protein needs. Pig production offers higher opportunities for achieving sustainable animal protein accessibility and economic gains, reducing poverty and protein shortages due to their unique characteristics when compared with other animals used as food [12]. However, despite the profitability of piggery, diversifying challenges increased from numerous swine diseases, poor biosecurity, and disease mitigation strategies to the detriment of the enterprise [13]. Also, the possibility of growing productivity is debarred by the excessive loss of animals to various diseases [14–16]. This problem is made worse because there is not enough information about how and where different diseases and pathogens that cause them affect production, especially in sub-Saharan Africa [9,17].

To date, many PCV2 strains from pigs of various health statuses have been widely documented in different countries of the world [18–23]. However, there is a paucity of information on the detection and molecular characteristics of the virus currently circulating within the swine herds in Nigeria. A recent report asserted that there is gross neglect in determining the presence and prevalence of PCV2 in swine herds of the majority of pig-producing sub-Sahara African countries; the study further underscored the observable poor awareness about the viral agent and the numerous diseases associated with it among pig farmers in the entire region [9]. Therefore, this research aims to determine the farmers’ level of awareness of PCV2, its associated diseases, and its implications in the southwestern region of Nigeria. This is very important because it is expected to provide baseline data for detailed epidemiological studies shortly and serve as a wake-up call for researchers and and other stakeholders in the pig farming industry in Nigeria.

**Table 1.** Porcine circovirus-associated diseases and their clinical features.

Name of disease	Type of pigs usually affected	Other implicated or coinfecting pathogens	Clinical signs
Postweaning multisystemic wasting syndrome	Nursery, growing, and adult pigs	PPV1, porcine reproductive and respiratory syndrome virus (PRRSV), *Mycoplasma hyopneumoniae*, etc.	Wasting, weight loss, pallor of the skin, ill thrift, enlarged lymph nodes, diarrhea, and respiratory distress.
Porcine dermatitis and nephropathy syndrome	Nursery, growing, and adult pigs	PRRSV and some bacteria such as* Actinobacillus pleuropneumoniae*, *Escherichia coli*, *Haemophilus parasuis *	Severe weight loss, depression, anorexia, mild pyrexia, stiff-gait, dark –red papules and macules on skin, majorly at the hind limbs and perineal region.
Porcine respiratory disease complex	Common in 8–26-week-old pigs	PRRSV, swine influenza virus (SIV), *A. pleuropneumoniae*, *P. multocida*, and *M. Hyopneumoniae*	Respiratory disorders, slow growth, Pneumonia, anorexia, dyspnea, fever, lethargy, cough and decreased feed efficiency.
Reproductive failure	Sows	PPV1, encephalomyocarditis virus (EMCV), Aujeszky’s disease virus (ADV), PRRSV	Late term abortions, stillbirths, premature piglets birth, fetal mummification, mid-gestation abortion, early embryonic death and regular return-to-estrus.
Granulomatous enteritis	Common in 8–16-week-old pigs	*Lawsonia intracellularis*	Diarrhea, unique lesions in Peyer patches.
Exudative epidermitis	Piglets of 5–35 days old, occasional mild cases in adult pigs	*Staphylococcus hyicus*, PPV1	Skin with an odoriferous exudate of serum and sebum, resulting to a dirty, moist and greasy appearance.

Source: [4, 6,7].

## MATERIALS AND METHODS

### Ethical approval

All ethical standards for research were followed in this study. No animal or human samples or related tests were involved in this study. Signed consent was obtained from the presidents of the pigs’ farmers association and the informed consent was obtained verbally from all the participants who were briefed about their rights to withdraw from the study. This is in line with the Declaration of Helsinki, which was signed in 2001.

### Location

**Figure 1.**
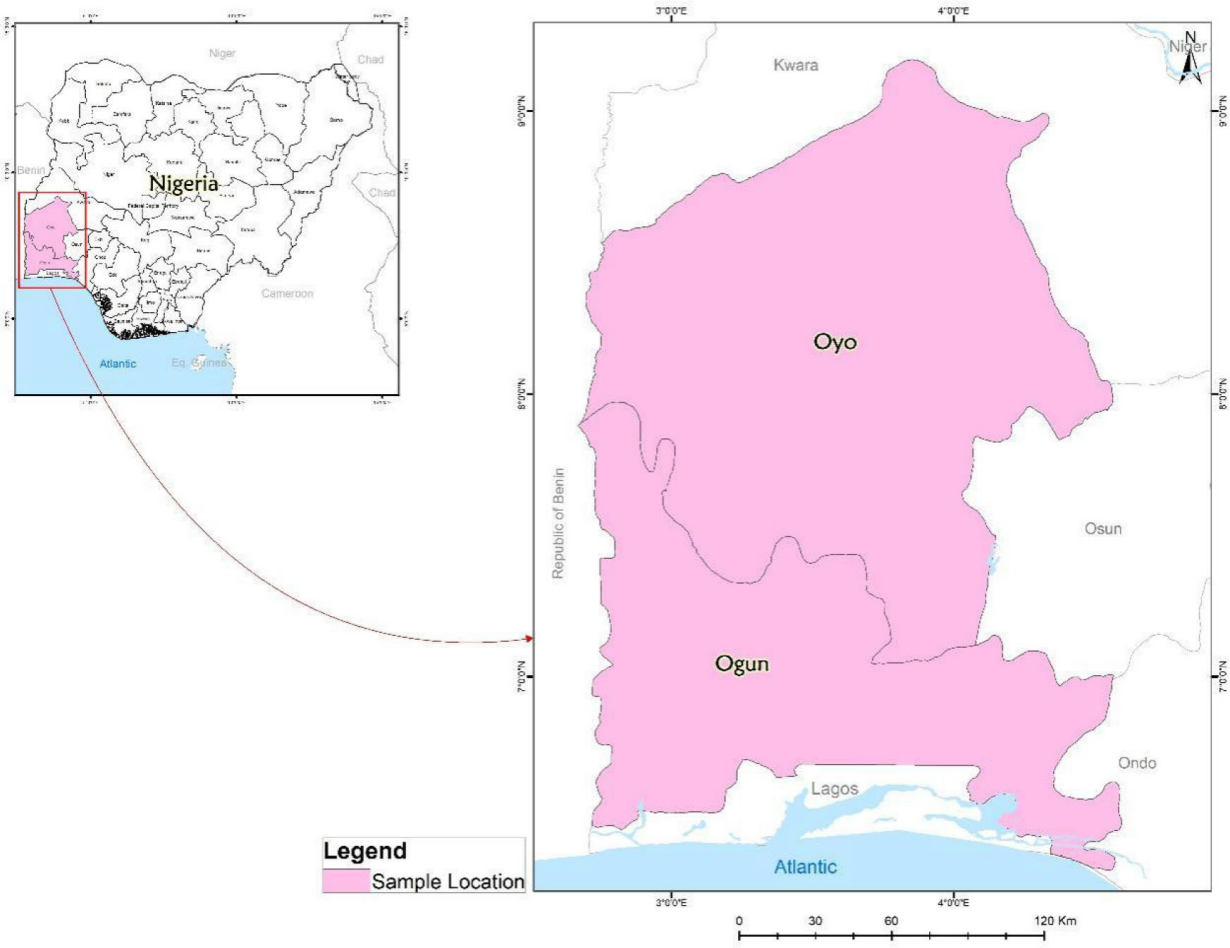
Spatial representation of Oyo and Ogun states, Southwest Nigeria.

This study was carried out in two states, namely Oyo and Ogun states, located in the southwestern geopolitical zone of Nigeria (Fig. 1). On the northern axis, Oyo state is bordered to the north by Kwara state, while Osun state is located on the eastern side. Ogun state and the Republic of Benin are situated on the western axis of the state. The projected human population was 7,840,864 as of 2016. Furthermore, Ogun state shares borders with Ondo state on the eastern axis; Oyo and Osun states on the north; Lagos state and the Atlantic Ocean on the south; and the Republic of Benin on the west, making it an essential gateway to the expansive markets of the Economic Community of West African States [24,25]. Both states were chosen for the study as they have a relatively high livestock population, including poultry, catfish, and pigs. All commercial pig farmers in the states of Oyo and Ogun were welcome to participate in this study.

### Research study design and determination of sample size

A cross-sectional survey was conducted to determine PCV2 awareness among pig farmers in Oyo and Ogun states, Southwest Nigeria. All pig farmers in these states were eligible to participate. Before the survey, meetings were held with the leaders of both chapters’ pig farmers’ associations, and signed consent was obtained. Individual pig farmers were recruited during the association’s monthly meetings, and verbal permission from volunteers was obtained. A nonprobabilistic sampling selection was conducted because the recruitment of participants was based on individuals’ availability and readiness to participate in the study. Involvement in the study was voluntary, with no penalty for anyone who refused to participate; personal details were not taken, and information from participants was strictly treated as confidential. All the participants were informed of their right to leave the study at any stage following the World Medical Association’s Declaration of Helsinki.

The sample size used in this study was determined by assuming that the level of awareness about PCV2 among the participants was 50%, with an absolute precision of 95% confidence interval and an acceptable error of 5%. With the use of WinEpi v.2.0, it was estimated that there were 385 participants who were split evenly between the states.

### Questionnaire design, pretest, and administration

The farm manager’s questionnaire consisted of 2 sections and 10 questions altogether. Information on the farm characteristics, such as location, type, and number of livestock present on farm premises and opening hours, were gathered. The other section comprised questions on awareness of PCV2 and other associated diseases, especially PMWS. Participants also provided data on observed symptoms or signs associated with PCV2/PMWS. Also, the preferred sources of creating awareness of the disease were suggested by farmers. A questionnaire was pretested among five pig farmers who were not included in this study. It took an average of 10 minutes to get the questionnaire filled in.

### Data analysis

Data processing was carried out using Microsoft Excel 2007, and the descriptive statistics were evaluated for all variables in the questionnaire as frequencies and proportions. To determine the level of awareness for PCV2/PMWS, binary responses were recorded as follows: “Yes” and accurate responses were scored “1,” and “No,” “not sure,” and “inaccurate responses” were scored as “0.” The scoring system ranged from 0 to 6, and all scores were converted to 100%. The cumulative score range was further re-categorized as “poor” (≤50%) and “satisfactory” (>50%). Graphic presentations were made using Microsoft Excel 2007.

## RESULTS

### Participation and farm characteristics

**Figure 2.**
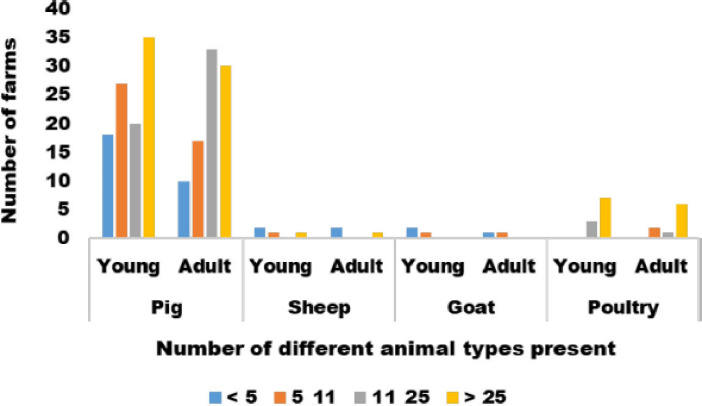
Number of farms and various animal species present.

A total of 111 farms participated out of 385 required (total response rate: 28.8%). All the participants who filled out the questionnaire had pigs raised on the premises (100%). Mixed farming was less practiced (15.3%). Other species present on pig farms were sheep, goats, and poultry. Figure 2 shows the number of farms and the number of young and adult animals of various species present.

### Level of awareness and recognition of clinical signs or symptoms associated with PCV2/PWMS

Table 2 summarizes participants’ knowledge of PCV2/PMWS in Southwest Nigeria. Out of the total pig farmers that participated, 89 (79.2%, 95% CI = 70.8–85.8) were unaware of PCV2, while 46 (41.4%, 95% CI = 32.7–50.7) indicated they had heard about PMWS. Overall, 67 (64.4%) participants indicated they were unsure if a vaccine was available to prevent the disease, while 35 (34.0%) were aware that the condition impacts reproduction. The level of awareness was generally poor, with an average score of 1.43 (SD ± 1.25; 23.9%). Less than 23% (25/111) of the participants fell within the category of having a good level of awareness.

### Perceived clinical signs/symptoms of PCV2/PMWS by pig farmers are described

Table 3 presents all the signs/symptoms listed in the questionnaire, which were observed and perceived by pig farmers as associated with PCV2/PMWS. Most people noticed weakness (85.2%), weight loss (84.7%), dermatitis (83.2%), diarrhea (83.2%), slow growth (78.4%), paleness (73.8%), swollen lymph nodes (66.6%), abortion/stillbirth (60.8%), trouble breathing (56.5%), and jaundice (25.7%).

### Preferred communication channels for creating awareness about this disease among pig farmers

**Figure 3.**
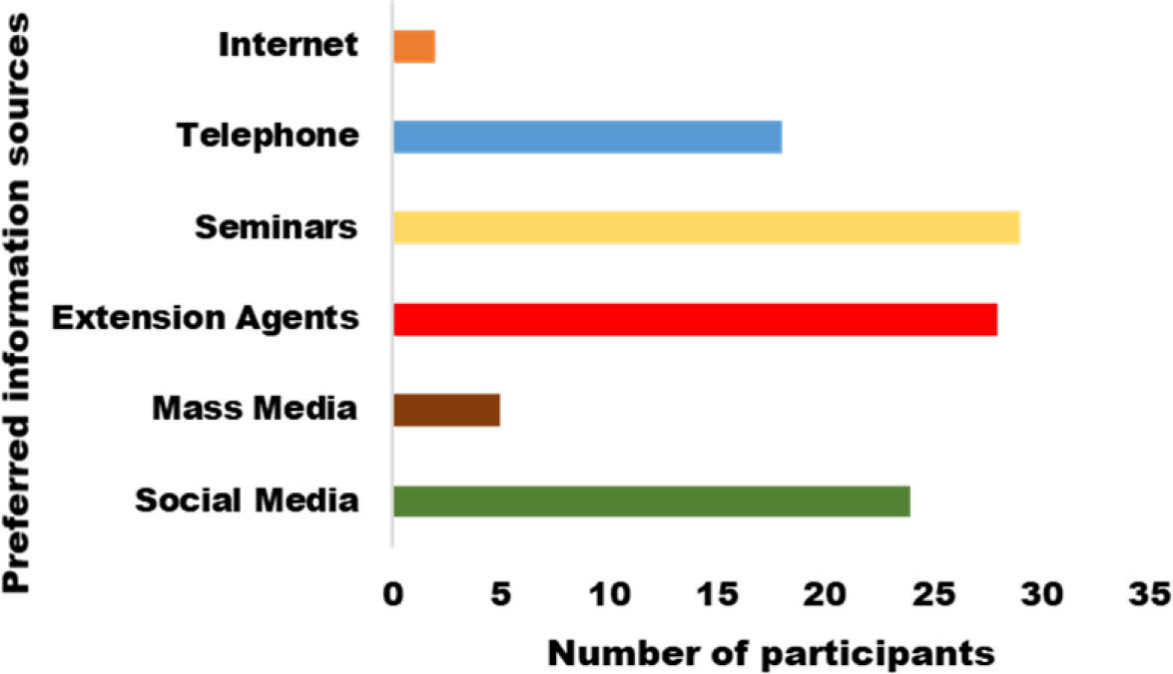
Preferred communication channels by pig farmers for literacy programs on PCV2/PMWS.

Most of the participants said that seminars, extension services (especially from veterinary and agricultural extension officers), social media (WhatsApp and YouTube), and mobile phones (through calls or text messages) were good ways to learn more about PCV2/PMWS (Fig. 3).

**Table 2.** Level of awareness of PVC2/PMWS among pig farmers in Southwest Nigeria.

Variables	Responses	Proportions (%)	95% CI
I have heard of porcine circovirus (PCV2) before (*n *= 111).	Yes No/Not sure	23 (20.7) 88 (79.3)	14.2–29.2 70.8–85.8
I have heard of postweaning multisystemic wasting syndrome. (*n *= 111).	Yes No/Not sure	46 (41.4) 65 (58.6)	32.7–50.7 49.3–67.3
PCV2 is an emerging swine pathogen of great economic importance (*n *= 101).	Yes No/Not sure	19 (18.8) 82 (81.2)	12.3–27.6 72.4–87.7
Disease treatable by antibiotics (*n *= 105).	Yes No/Not sure	65 (61.9) 40 (39.1)	52.3–70.6 29.4–47.7
Vaccines available for the prevention of this disease (*n *= 104).	Yes No/Not sure	37 (35.6) 67 (64.4)	27.0–45.1 54.9–73.0
PCV2/PMWS has reproductive implications (*n *= 103).	Yes No/Not sure	35 (34.0) 68 (66.0)	25.5–43.6 56.4–74.5

*n *= number of respondents.

**Table 3.** Perceived clinical signs/symptoms associated with PCV2/PMWS by pig farmers in Southwest Nigeria.

Clinical signs associated with PCV2/PMWS	Yes (%)	No (%)	Very frequently (%)	Frequently (%)	Less frequently (%)
Paleness	62 (73.8)	22 (36.2)	0 (0.0)	3 (9.1)	30 (90.9)
Diarrhea	79 (83.2)	16 (16.8)	3 (7.3)	8 (19.5)	30 (73.2)
Difficulty in breathing	48 (56.5)	37 (43.5)	2 (5.5)	6 (16.6)	28 (77.7)
Weight loss	83 (84.7)	15 (15.3)	3 (7.3)	10 (24.4)	28 (68.3)
Retarded growth	58 (78.4)	16 (21.6)	4 (11.8)	6 (17.6)	24 (70.6)
Weakness	69 (85.2)	12 (14.8)	2 (6.6)	2 (6.6)	26 (86.8)
Enlarged lymph nodes	20 (66.6)	30 (33.4)	0 (0.0)	2 (13.3)	13 (86.7)
Jaundice	18 (25.7)	52 (74.3)	0 (0.0)	1 (12.5)	7 (87.5)
Dermatitis	75 (83.3)	15 (16.7)	1 (2.7)	9 (24.3)	27 (73.0)
Abortions/stillbirth	56 (60.8)	36 (39.2)	1 (3.2)	3 (9.7)	27 (87.1)

## DISCUSSION

PCV2, the principal etiologic agent of the PMWS, could surreptitiously cause a significant economic loss in the pig business, especially in areas where the viral infection and its accompanying diseases are poorly understood [8,9,26]. This study investigates the awareness of pig farmers in the southwestern region of Nigeria on PCV2 and its related diseases. Our findings indicated that the awareness level of pig farmers was generally low, with relatively few participants (precisely 21.8%) having some level of awareness of PCV2 and less than 50% being aware of PMWS, which is the major clinical outcome of PCV2 infection. This finding further corroborates a survey in some South African pig farms in which 85.7% of the farmers had no prior knowledge of PCV2 and its related diseases [22]. The importance of an immediate and thorough public awareness campaign on some neglected transboundary infectious agents and diseases cannot be overstated. This is because it has become crystal clear that quite a number of them are very much within the African region, even though they are still regarded as non-notifiable in many countries [9,27].

Pig production is an important asset and survival mechanism for rural and semi-urban farmers, and it may provide an opportunity to improve their quality of life by increasing cash income from sales and improving nutrition [27,28]. However, inevitable setbacks exist due to the paucity of information on the prevalence of various diseases, including the PCV2 infections, and their negative impacts on piggery, particularly in developing countries [17,29], where animals owned by low-income farmers are more susceptible to diseases [30].

Notably, a little over one-third of the farmers were aware of the availability of vaccines to prevent PCV2 infections among their animals in the study area, whereas about two-thirds of the respondents had a notion that diseases due to PCV2 infections were treatable with antibiotics. This finding is highly significant as it could be one of the pertinent reasons for the indiscriminate application of antibiotics in pig rearing in the studied region [13]. Due to a poor level of awareness, it is perceived by this study that many farmers are resorting to the use of antibiotics in treating seemingly PCV2 infection symptoms (including diarrhea, stunting, dermatitis, etc.) based on their crude experience without addressing the actual cause of the symptoms. This claim could be backed up by the fact that most farmers said they had seen many symptoms related to PCV2/PMWS on their farms.

Vaccination remains the most cost-effective process that could be employed for the prevention of livestock diseases. Generally, the process is secure, effective, and has little or no serious adverse effects [31]. Vaccines are advantageous in conferring long-term prophylaxis, helping to avoid infectious diseases and illnesses that may be costlier to manage compared to the financial requirement of vaccination. It is imperative to implement an effective vaccination regimen in livestock farming for effective management and optimal profits. It boosts immunity, reduces the effects of subclinical diseases, curbs the spread of infectious diseases, and plays a crucial role in eradicating diseases [31]. The same thing applies to PCV2, as many commercial vaccines are currently available that could be used in a bid to forestall or mitigate PCV2 infection in pigs [32,33]. However, farmers need to know the effective methods of administering PCV2 vaccines [34,35]. Although it has been stipulated that a mass vaccination process observed for 12 months could not bring about the eradication of the viral pathogen, an appreciable success has been recorded with a notable decrease in the prevalence of PCV2 [8,32]. So, it is essential that the government agencies in charge of agriculture in Nigeria and other African countries review the fact that PCV2 is not a disease that needs to be reported, take the lead in letting farmers know about the pathogen, and stress the need for effective vaccination instead of the overuse of antibiotics.

Also, this study shows that most pig farmers are experiencing the clinical signs and symptoms associated with PCV2/PMWS. These signs and symptoms range from growth retardation, weight loss, paleness, wasting, stillbirths, abortions, weakness, jaundice, anemia, diarrhea, and enlarged lymph nodes. The most prevalent signs and symptoms, in decreasing order, were weight loss, diarrhea, dermatitis, weakness, and paleness. Weight loss or wasting is a common clinical symptom of PMWS [36]. In weaner and finishing pigs, the wasting syndrome increases death rates and lowers the daily weight gain, resulting in an unimaginable economic loss to farmers. Again, it could also be observed that more than half of the farmers affirmed the occurrence of abortion among their sows. Late-term abortions and stillbirth have been seen to occur in sows infected with PCV2 [37,38]. Even though the signs and symptoms could be caused by something other than PCV2 in pigs, the viral pathogen should be seen as the most likely cause and should get the attention it needs.

## CONCLUSION

This study has further underscored the need for aggressive molecular epidemiology and surveillance of PCV and circulating genotypes in Nigerian pigs. This will help reveal swine herds’ true and current status in the country regarding the pathogen. This has become imperative as it could be seen from all indications that the viral agents might unknowingly ravage many herds in the country. To curb the spread of PCV2 diseases in pigs, proper management strategies that include improving sanitary measures and treating bacterial and viral cofactors associated with the diseases should be implemented. Also, there is a need for a countrywide rollout of vaccination programs as part of a national effort to control viral agents. However, all of these will only be possible if awareness about PCV2 and its related diseases increases through active research on the virus. 

## List of Abbreviations

Kb, kilobytes; nt, Nucleotide; PCV2, Porcine circovirus type 2; PCVADs, Porcine circovirus associated diseases; PMWS, Postweaning Multisystemic Wasting Syndrome; SDGs, Sustainable Development Goals; UN, United Nations.

## References

[1] Walker PJ, Siddell SG, Lefkowitz EJ, Mushegian AR, Dempsey DM, Dutilh BE (2019). Changes to virus taxonomy and the International Code of Virus Classification and Nomenclature ratified by the International Committee on Taxonomy of Viruses (2019). Arch Virol.

[2] Finsterbusch T, Mankertz A (2009). Porcine circoviruses—small but powerful. Virus Res.

[3] Allan GM, Ellis JA (2000). Porcine circoviruses: a review. J Vet Diag Invest.

[4] Opriessnig T, Xiao CT, Gerber PF, Halbur PG (2014). Identification of recently described porcine parvoviruses in archived North American samples from 1996 and association with porcine circovirus associated disease. Vet Microbiol.

[5] Palinski R, Piñeyro P, Shang P, Yuan F, Guo R, Fang Y (2017). A novel porcine circovirus distantly related to known circoviruses is associated with porcine dermatitis and nephropathy syndrome and reproductive failure. J Virol.

[6] Chae C (2005). A review of porcine circovirus 2-associated syndromes and diseases. Vet J.

[7] Segalés J (2011). Porcine circovirus type 2 (PCV2) infections: clinical signs, pathology and laboratory diagnosis. Virus Res.

[8] Alarcon P, Rushton J, Wieland B (2013). Cost of post-weaning multi-systemic wasting syndrome and porcine circovirus type-2 subclinical infection in England–an economic disease model. Prev Vet Med.

[9] Afolabi KO, Iweriebor BC, Okoh AI, Obi LC (2017). Global status of porcine circovirus type 2 and its associated diseases in sub-Saharan Africa. Adv Virol.

[10] Mistry B (2020). Pig farm industry and opportunities in Nigeria, 2017. https://www.danishfarmersabroad.dk/wp-content/uploads/2017/01/Nigeria-Presentation-to-DFA-18jan2017.

[11] UNDP (2020). The sustainable development goals report, 2017.

[12] Uddin I, Osasogie D (2017). Constraints of pig production in Nigeria: a case study of Edo Central Agricultural Zone of Edo State. Asian Res J Agric.

[13] Adebowale OO, Adeyemo FA, Bankole N, Olasoju M, Adesokan HK, Fasanmi O (2020). Farmers’ Perceptions and drivers of antimicrobial use and abuse in commercial pig production, Ogun State, Nigeria. Int J Environ Resour Public Health.

[14] Muhanguzi D, Lutwama V, Mwiine FN (2012). Factors that influence pig production in Central Uganda-Case study of Nangabo Sub-County, Wakiso district. Vet World.

[15] Muwonge A, Johansen TB, Vigdis E, Godfroid J, Olea-Popelka F, Biffa D (2012). *Mycobacterium bovis* infections in slaughter pigs in Mubende district, Uganda: a public health concern. BMC Vet Res.

[16] Ndyomugyenyi EK, Kyasimire J (2015). Pig production in Kichwamba Sub-county, Rubirizi district, Uganda. Livest Res Rural Dev.

[17] Dione MM, Ouma EA, Roesel K, Kungu J, Lule P, Pezo D (2014). Participatory assessment of animal health and husbandry practices in smallholder pig production systems in three high poverty districts in Uganda. Prev Vet Med.

[18] Grierson SS, King DP, Wellenberg GJ, Banks M (2004). Genome sequence analysis of 10 Dutch porcine circovirus type 2 (PCV-2) isolates from a PMWS case-control study. Res Vet Sci.

[19] Knell S, Willems H, Hertrampf B, Reiner G (2005). Comparative genetic characterization of porcine circovirus type 2 samples from German wild boar populations. Vet Microbiol.

[20] Wen L, Guo X, Yang H (2005). Genotyping of porcine circovirus type 2 from a variety of clinical conditions in China. Vet Microbiol.

[21] Jeoung HY, Lim SI, Kim JJ, Cho YY, Kim YK, Song JY, Hyun BH, An DJ (2015). Serological prevalence of viral agents that induce reproductive failure in South Korean wild boar. BMC Vet Res.

[22] Afolabi KO, Iweriebor BC, Obi LC, Okoh AI (2017b). Molecular detection of porcine circovirus type 2 in swine herds of Eastern Cape Province, South Africa. BMC Microbiol.

[23] Afolabi KO, Iweriebor BC, Obi LC, Okoh AI (2019). Genetic characterization and diversity of porcine circovirus type 2 in non‐vaccinated South African swine herds. Transb Emerg Dis.

[24] Obayelu AE, Ogunmola OO, Sowande OK (2017). Economic analysis and the determinants of pig production in Ogun State, Nigeria. Agric Trop Subtrop.

[25] Adebowale OO, Adeyemo OK, Awoyomi O, Dada R, Adebowale O (2016). Antibiotic use and practices in commercial poultry laying hens in Ogun State Nigeria. Rev d’elev Med Vet Pays Trop.

[26] Rajesh JB, Rajkhowa S, Dimri U, Prasad H, Sarma K (2020). Need of alertness on porcine circovirus 2 in North East India. Int J Vet Sci Res.

[27] Mokoele JM, van Rensburg LJ, Van Lochem S, Bodenstein H, Du Plessis J, Carrington CA (2015). Overview of the perceived risk of transboundary pig diseases in South Africa. J South Afr Vet Assoc.

[28] Ironkwe MO, Amefule KU (2008). Appraisal of indigenous pig production and management practices in Rivers State, Nigeria. J Agric Social Res.

[29] Bisimwa PN, Wasso DS, Bantuzeko F, Aksanti CB, Tonui R, Birindwa AB (2021). First investigation on the presence of porcine parvovirus type 3 in domestic pig farms without reproductive failure in the Democratic Republic of Congo. Vet Anim Sci.

[30] Swai ES, Lyimo CJ (2014). Impact of African Swine fever epidemics in smallholder pig production units in Rombo district of Kilimanjaro, Tanzania. Livest Res Rural Dev.

[31] Roth JA (2011). Veterinary vaccines and their importance to animal health and public health. Proc Vaccinol.

[32] Beach NM, Meng XJ (2012). Efficacy and future prospects of commercially available and experimental vaccines against porcine circovirus type 2 (PCV2). Virus Res.

[33] Park KH, Oh T, Yang S, Cho H, Kang I, Chae C (2019). Evaluation of a porcine circovirus type 2a (PCV2a) vaccine efficacy against experimental PCV2a, PCV2b, and PCV2d challenge. Vet Microbiol.

[34] Afghah Z, Webb B, Meng XJ, Ramamoorthy S (2017). Ten years of PCV2 vaccines and vaccination: is eradication a possibility?. Vet Microbiol.

[35] Franzo G, Segalés J (2020). Porcine circovirus 2 genotypes, immunity and vaccines: multiple genotypes but one single serotype. Pathogens.

[36] Um H, Yang S, Oh T, Cho H, Park KH, Suh J, Chae C (2021). Veterinary medicine and science (VMS3-2021-Jun-0446). Vet Med Sci.

[37] Mak CK, Yang C, Jeng CR, Pang VF, Yeh KS (2018). Reproductive failure associated with coinfection of porcine circovirus type 2 and porcine reproductive and respiratory syndrome virus. Can Vet J.

[38] Tibary A (2021). Abortion in pigs. MSD veterinary mannual. Merck & Co., Inc., Kenilworth, NJ,.

